# Zinc is an important inter-kingdom signal between the host and microbe

**DOI:** 10.1186/s13567-021-00913-1

**Published:** 2021-03-04

**Authors:** Pengpeng Xia, Siqi Lian, Yunping Wu, Li Yan, Guomei Quan, Guoqiang Zhu

**Affiliations:** 1 College of Veterinary Medicine (Institute of Comparative Medicine), Yangzhou University, Yangzhou, 225009 China; 2 Jiangsu Co-Innovation Center for Prevention and Control of Important Animal Infectious Diseases and Zoonoses, Yangzhou, 225009 China; 3 Joint International Research Laboratory of Agriculture and Agri-Product Safety of Ministry of Education of China, Yangzhou University, Yangzhou, 225009 China

**Keywords:** Zinc ion (Zn^2+^), Inter-kingdom signal, Microbial pathogenesis, Immune response, Zinc deficiency

## Abstract

Zinc (Zn) is an essential trace element in living organisms and plays a vital role in the regulation of both microbial virulence and host immune responses. A growing number of studies have shown that zinc deficiency or the internal Zn concentration does not meet the needs of animals and microbes, leading to an imbalance in zinc homeostasis and intracellular signalling pathway dysregulation. Competition for zinc ions (Zn^2+^) between microbes and the host exists in the use of Zn^2+^ to maintain cell structure and physiological functions. It also affects the interplay between microbial virulence factors and their specific receptors in the host. This review will focus on the role of Zn in the crosstalk between the host and microbe, especially for changes in microbial pathogenesis and nociceptive neuron-immune interactions, as it may lead to new ways to prevent or treat microbial infections.

## Introduction

Zinc (Zn) is the second most abundant trace metal in living organisms and is involved in numerous aspects of life, including but not limited to DNA replication, transcription, protein synthesis, cell proliferation, apoptosis, and signal transduction. Both animals and microbes cannot naturally produce Zn, and the strategy for withholding and using Zn is vital to their survival and development [1, 2]. Within the expected concentration range, animals and microbes can regulate their internal Zn concentrations to maintain physiological metabolism [3, 4]. However, excess amounts of zinc ions (Zn^2+^) will lead to some cytotoxic effects, while zinc deficiency results in disruption of normal biological activities [3, 5].

Numerous studies have shown that zinc deficiency in animals leads to retarded growth, impaired immunity, and severe pathological changes in the body [4]. However, the host uses a similar strategy to reduce the zinc concentration in a single location against bacterial infection, which is called “nutritional immunity”, limiting the growth and virulence of pathogenic bacteria [6]. During the infectious process, some zinc-sequestering proteins, such as calprotectin, are expressed and recruited to the infection site to limit zinc acquisition by bacteria [7]. To survive in the host and succeed in competing with commensal microbes, pathogenic bacteria must maintain a steady state of zinc usage by controlling zinc distribution with zinc transporters [2, 3, 8]. The presence of zinc transporters in pathogenic bacteria also contributes to maintaining zinc homeostasis and virulence [2, 9]. Moreover, zinc acts as a direct or indirect regulator to affect the communication between nociceptor neurons and immune systems in the host, modulating the inflammatory response and host defence against bacterial infection [10–12].

Although nutritional immunity is termed a direct and effective antibacterial immune response, these mechanisms are not well described in the case of virus infection; for example, calprotectin did not to have a proven antiviral effect in published papers. However, zinc has been noted as a direct antiviral drug, as well as an antiviral immune stimulator and an indispensable component for the replication of many viruses [13–15]. Thus, zinc is likely to be a potential inter-kingdom signal between the host and microbes. In this review, we describe recent advances in understanding the role of zinc, similar to host hormones and quorum sensing (QS) of bacteria [16, 17], in modulating the communication between pathogenic microbes and their hosts.

## Zinc homeostasis in bacteria

**Figure 1:**
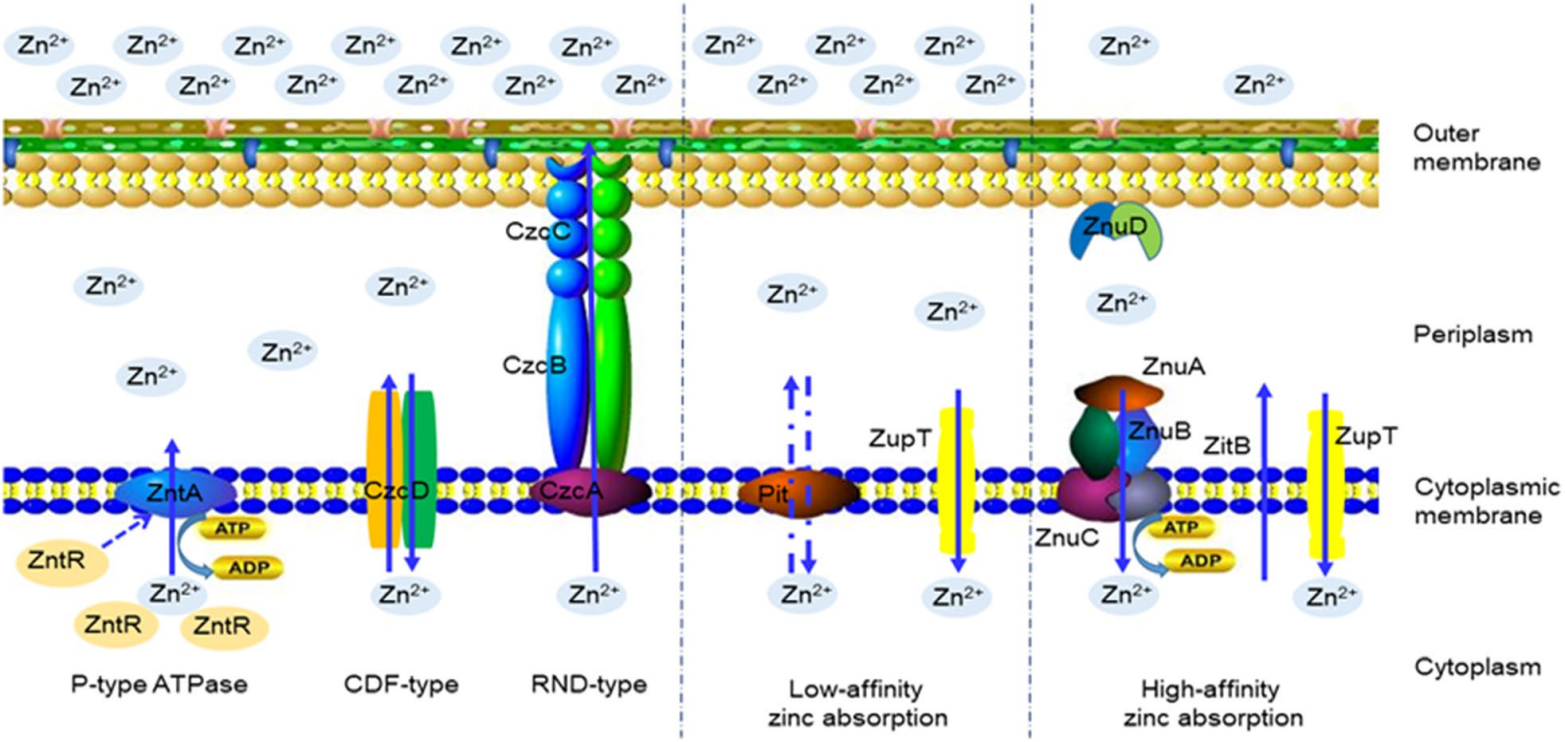
**Zinc transporter systems in bacteria** [2, 9, 19–22]**.** Under zinc-replete conditions, membrane transport based on the P-type ATPase, CDF-type and RND-type families participates in zinc distribution. They work together to maintain zinc homeostasis in the cell. With sufficient zinc supplementation, bacteria control the cellular zinc concentration by a low-affinity absorption system, such as Pit and ZupT. Under zinc-limiting conditions, transporters of the high-affinity absorption system are induced and used to recruit Zn^2+^ against zinc deficiency, including ZnuACB, ZitB, ZnuD, etc.

In addition to being a cofactor for some bacterial proteins, zinc ions are required for DNA repair, enzymatic reactions, responses to oxidative stress, and regulatory roles in other physiological processes in bacteria [2, 18]. Studies have shown that at least four kinds of zinc transport systems in bacteria, including two uptake transporters, ZnuACB and ZupT, and two export proteins, ZntA and ZitB, maintain zinc concentrations in the cell [2, 19]. As shown in Figure 1, under the pressure of a high Zn^2+^ concentration, the protein expression of P-type ATPase, cation diffusion facilitator (CDF) family of membrane transport, and resistance nodulation division (RND)-type efflux pumps are induced and protect bacteria from zinc poisoning [20, 21]. When zinc supplementation is sufficient for basic needs, bacteria synthesize low-affinity absorption systems to control zinc transport, such as the inorganic phosphate transport (Pit) system, which catalyses the rapid exchange of zinc via chemical osmotics [21]. Under zinc-limiting conditions, bacteria absorb and transport Zn^2+^ against zinc deficiency via a high-affinity ZnuACB uptake transporter [22, 23].

The ZnuACB transporter usually consists of three parts, including the periplasmic Zn^2+^-binding protein ZnuA, the inner membrane channel protein ZnuB and the ATP enzyme protein ZnuC, which is responsible for providing energy for Zn^2+^ transport [21]. ZinT and ZnuA have been shown to cooperate in periplasmic zinc recruitment, and the role of ZinT in the zinc uptake process is dependent on the presence of ZnuA [23, 24]. These genes are regulated by Zur, which not only blocks the binding of RNA polymerase and inhibits the expression of ZnuACB by Zn_2_Zur (or Zn_4_Zur) dimer protein but also regulates the mobilization of zinc through ribosomal proteins and leads to ZupT transporting Zn^2+^ rather than ZnuACB [18, 20]. Both ZupT and ZnuACB are necessary for bacteria to grow under zinc limitation; although ZnuACB usually obscures the role of ZupT, it also contributes to zinc uptake by *Escherichia coli* (*E. coli*) and *Salmonella* at a zinc concentration of 10 mM [25, 26].

In recent reports, the ZnuACB transport system is the most important zinc transport system in many bacteria [2, 27, 28]. *znuA* gene deletion can significantly reduce the adhesion of *E. coli* O157:H7 and *Campylobacter jejuni* to host cells [24, 29] and attenuate the pathogenicity of *Brucella abortus*, *Salmonella* enteritidis, and *Pasteurella multocida* to susceptible animals [27, 30]. The motility and biofilm formation of uropathogenic *E. coli* (UPEC) CFT073*△znuB* decreased compared with that of the wild-type strain [28]. After the *znuACB* gene was deleted, the virulence of *Salmonella* Typhimurium (STm) was weakened, but the existence of a ZnuACB transport system allowed the bacteria to resist calprotectin-mediated Zn^2+^ chelation and benefit from competition with host microflora [8, 27]. Our study also found that the ZnuACB system of enterotoxigenic *E. coli* (ETEC) F4ac plays an important role in maintaining the formation of biofilms and adherence to porcine small intestinal epithelial cells in vitro under zinc deficiency [31]. Although deletion of the *zur* gene does not affect the virulence of *Staphylococcus aureus* (*S. aureus*) and *Mycobacterium tuberculosis* (*M. tuberculosis*), it decreases the mortality of mice infected with STm [32–34]. Under zinc-limiting conditions, the growth of *E. coli* Nissle 1917 did not weaken due to the deletion of zinc transporter genes, and the bacteria utilize yersiniabactin to scavenge zinc to resist calprotectin-mediated zinc sequestration in the inflamed gut [35]. The newly discovered TonB-dependent outer membrane protein receptor ZnuD in *Neisseria meningitidis* regulates the absorption of zinc and haem and can induce antibodies combined with vesicles to infect guinea pigs and activate complement-mediated cytotoxicity to kill bacteria [36, 37]. Thus, in addition to regulating zinc concentrations in the cell, zinc transporters can also affect the expression of virulence factors and the pathogenicity of bacteria. It is likely to be an effective target for the development of new antibacterial drugs or vaccines.

## Zinc is an essential micronutrient for the host

Zinc sufficiency is crucial for the maintenance of zinc homeostasis and health in the host, while severe zinc deficiency induces apoptosis via the activation of the caspase pathway [38]. During this process, mitochondrial p53 mediates the nuclear translocation of apoptosis-inducing factor and endonuclease G and triggers subsequent cell death [38, 39]. Moreover, within the normal range, zinc can directly interfere with caspase and endonuclease activity and thereby suppress cell apoptosis [40]. In addition, it can effectively inhibit the accumulation of reactive oxygen species (ROS) in the body, reduce oxidative damage to proteins, and maintain cell membrane structure [41, 42].

Indeed, in the early stage of zinc insufficiency, cells upregulate the mRNA expression of the zinc-iron regulatory protein ZIP1-ZIP14 (Zrt, Irt-like protein/SLC39) and mediate zinc influx and intracellular redistribution to increase zinc uptake [43, 44]. Later, the mRNA expression of divalent metal transporter 1 (DMT1) is upregulated, and DMT1 cooperates with ZIP to maintain the stability of Zn^2+^ concentrations [45, 46]. On the other hand, with an excessive intake of zinc, cells increase zinc binding and zinc efflux by upregulating the mRNA expression of metallothionein (MT)-1 and the zinc transporter ZnT/solute-linked carrier 30 (ZIP/SLC30) family proteins ZnT1-ZnT10 [44, 47]. Thus, zinc transporters are critical to maintaining intracellular Zn^2+^ concentrations and are closely involved in zinc uptake and zinc efflux.

The absorption of Zn^2+^ by mammals mostly occurs in the distal small intestine, and the absorption process is mainly divided into four stages: intestinal cell uptake, mucosal cell transport, portal vein circulation, and endogenous zinc secreted back to intestinal cells [48]. Intestinal cells regulate intracellular Zn^2+^ concentrations mainly through exogenous zinc absorption and the efflux of endogenous zinc. Zinc transporters are also reported to be related to zinc homeostasis regulation and dynamic changes in intestinal epithelial cells [44, 47]. For instance, ZIP4 contributes to the differentiation and maintenance of Paneth cells, ZnT2 regulates antibacterial substance secretion in Paneth cells, and ZIP8 negatively regulates pro-inflammatory responses by promoting zinc efflux out of the organelles or extracellular zinc uptake into monocytes, macrophages, and pulmonary epithelial cells [47, 49]. ZIP10 inhibits apoptosis but has been implicated in gastric and colon cancer, while the ZIP6/ZIP10 heterodimer was required for zinc influx into cells to trigger the onset of mitosis and influence cell division progression [49, 50]. The loss of ZIP4 in the intestinal epithelium led to changes in mammalian rapamycin target protein (mTOR) signal transduction in villi and crypts and resulted in Paneth cell viability and functional disorders [51]. Moreover, an imbalance in Zn^2+^ homeostasis occurred in the intestine of ZIP7- and ZIP14-deficient mice, ZIP7 promoted the proliferation of crypt cells, and ZIP14 protected the structure of intestinal epithelial cells by maintaining stable expression and modification in tight junctions (TJs), such as phosphorylation of occludin proteins. The deletion of ZIP7 triggered endoplasmic reticulum (ER) stress in proliferative progenitor cells, resulting in a large amount of cell death, and ZIP14 deletion impaired the integrity of the intestinal epithelial barrier by reducing the expression of the zonula occludens (ZO)-1 and claudin-1 proteins [49, 52, 53].

The stability and catalytic activity of proteins is mostly promoted by metal binding, whereas incorrect metallization causes protein denaturation, enzyme inactivation, and even cell death. To avoid damage caused by insufficient zinc binding in proteins, cells have to control the expression or activity of zinc transporters to maintain a steady cellular zinc balance [49, 54]. Intracellular labile zinc is mostly harvested in organelles, including mitochondria, the ER, and the Golgi apparatus [55]. Even the accumulation of zinc ions leads to ER stress and sometimes reduces cell viability. These ions are essential for maintaining the activation of specific proteins and zinc homeostasis in life [42, 56]. For example, zinc ions stored in the ER of *Arabidopsis thaliana* will be triggered by the appropriate signal and released into the cytoplasm to cope with the redistribution of Zn^2+^ under conditions of zinc deficiency [57]. Yeast can effectively regulate intracellular zinc concentrations by changing the zinc proteome and the expression of zinc transporters at the transcriptional and posttranslational levels, especially in zinc-deficient environments [56].

Zinc transporter activity is also related to the zinc-sensing receptor protein ZnR/GPR39 (G Protein-Coupled Receptor 39) in intestinal epithelial cells [58]. The ZnR/GPR39 protein belongs to the G protein-coupled receptor (GPCR) family and mediates Zn^2+^-dependent signal transduction in keratinocytes, pancreatic cells, prostate cancer cells, salivary gland cells, neurons and bone cells [58, 59]. The GPR39 protein binds to Zn^2+^ through two histidine residues, His17 and His19, and the aspartic acid residue Asp313. It senses changes in Zn^2+^ concentrations but is not activated by Mn^2+^, Cu^2+^ or Fe^2+^ [58]. After activation by Zn^2+^, ZnR/GPR39 controls the absorption of Cl^−^ and reduces fluid loss during diarrhoea by upregulating potassium-chlorine cotransporter 1 in the basolateral colon [60]. It also mediates Gq-dependent downstream signal transduction, inducing increased expression of Na^+^/H^+^ exchanger (NHE), which is attributed to the recovery of the pH in the intestine [58, 60]. An acidic pH of 6.5 can have a profound effect on ZnR/GPR39 activity, leading to a loss of stimulation of the extracellular protein kinase (ERK1/2) or protein kinase B (Akt) pathway and reduced NHE activation [58]. Asp313 was proven to be the key residue for ZnR/GPR39 to sense extracellular pH changes; therefore, for survival, ZnR/GPR39 must maintain pH homeostasis in the intestine [61]. In human colon epithelial cancer cells (Caco-2), through the mitogen-activated protein kinase (MAPK) and phosphatidylinositol 3-kinase (PI3K) pathways, ZnR/GPR39 activated Zn^2+^-dependent mTOR/p70 ribosomal S6 protein kinase (p70S6K) and protein kinase C-ζ (PKCz), enhanced cell proliferation and differentiation, and promoted the abundant expression of ZO-1, occludin and E-cadherin to attenuate the colitis and intestinal damage caused by STm infections [59, 62].

## Zinc plays an important role in the host-pathogenic bacteria interaction

In normal organisms, to prevent the proliferation and infection of pathogens, host cells limit the concentration of intracellular free Zn^2+^ by zinc binding and efflux, while bacteria rely on an efficient zinc transport system to maintain zinc supplementation for proliferation and disruption of the host defence system to establish infection [9, 18, 27]. Therefore, there exists competition in zinc acquisition between the host and pathogenic bacteria, and gut health seems to be a good target for studying this competitive relationship.

**Figure 2:**
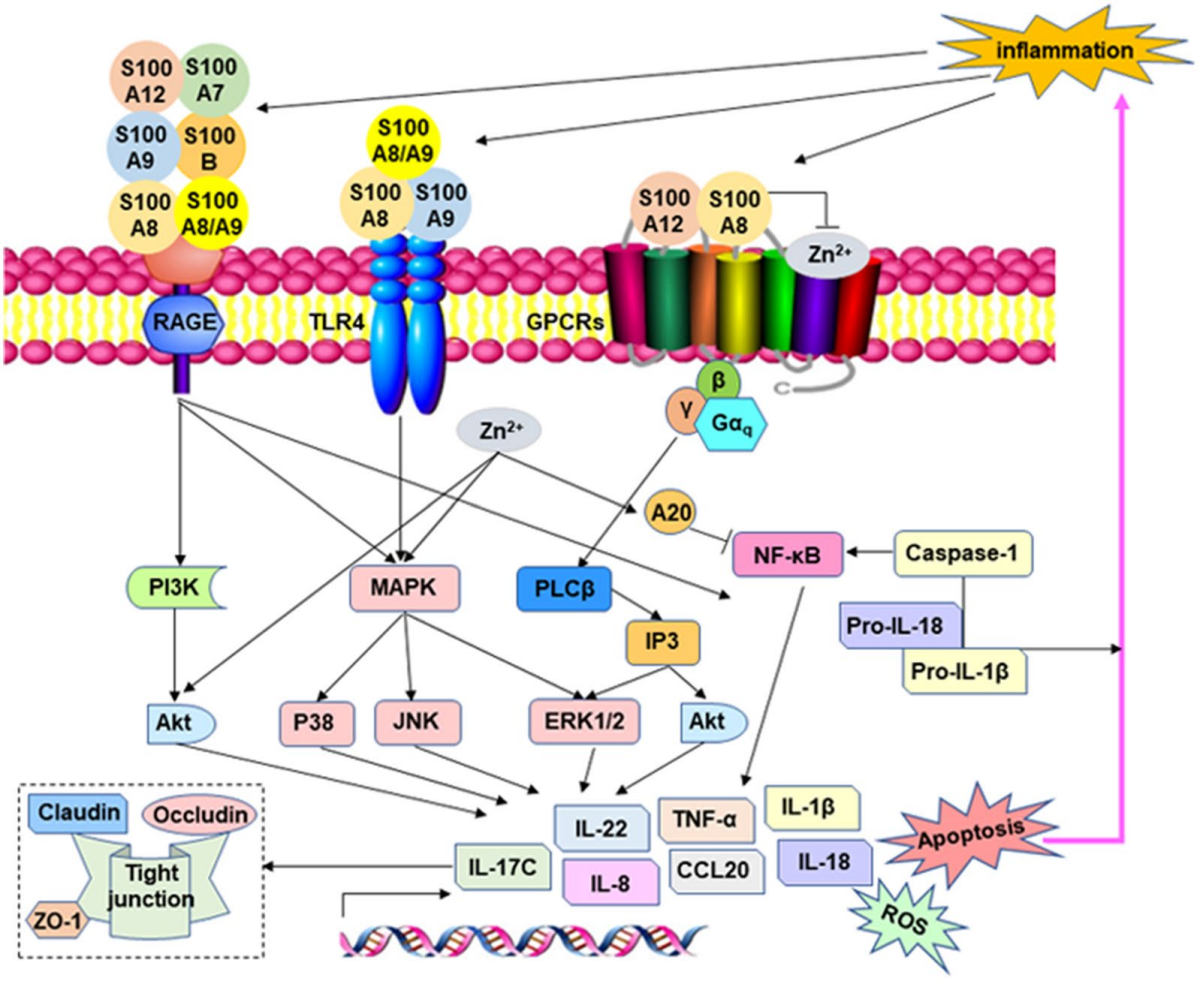
**Regulation of mucosal immune homeostasis in the intestine** [7, 71, 78, 108, 123, 133]**.** The dynamic change in competition in zinc acquisition between the host and pathogens is complicated. In addition to playing a role in enzymes and structural cofactors for the survival of all organisms, zinc also affects the activity of some transmembrane receptor proteins, including TLRs, which are responsible for the recognition of microbes or antigen molecules and the development and modulation of immune responses. Upon pathogen attack, some zinc-sequestering proteins are expressed and recruited to the infected site to chelate zinc ions and limit the growth and virulence of pathogenic bacteria, such as calprotectin, also known as the S100A8/S100A9 heterodimer, which is vital for strategic nutritional immunity. During the infectious process, the host also releases other S100 proteins, and then the binding of S100 proteins to cell surface receptors, such as TLR4, RAGE, and GPCRs, plays an important role in the regulation of inflammatory signal transduction. In turn, by regulating downstream signalling pathways, zinc can enhance the integrity of the intestinal mucosal barrier and reduce inflammation and diarrhoea caused by pathogenic infection.

The intestinal mucosal barrier is the first line of defence against pathogens and other stimulators in the gut (Figure 2) [44, 63]. The protective effect is determined by the integrity of intestinal epithelial cells, the stability of the mucus layer, and normal expression or distribution of the E-cadherin, occludin, claudins, and ZO proteins [47, 64]. It has been reported that *E. coli* infections can change the expression of TJ proteins in the host and induce cytoplasmic distribution of claudin-2, resulting in an increase in intestinal epithelial permeability and a rapid decrease in transepithelial electrical resistance [65]. Upregulation of claudin-2 is induced by interleukin (IL)-22, resulting in enhancement of epithelial permeability and water efflux, which facilitates *Citrobacter rodentium* (*C. rodentium*) infection clearance through diarrhoea [65, 66]. In fact, intestinal mucosal immune responses in the host are activated shortly after an infection, and during this process, a number of pro-inflammatory cytokines and chemokines are expressed and released, including monocyte chemoattractant protein-1, IL-8/CXCL8, and tumour necrosis factor (TNF)-β [67]. According to recent reports, IL-17 exerts anti-inflammatory effects in the intestine by regulating the secretion of secretory immunoglobulin A (sIgA). IL-17 promotes the secretion of chemokines, such as CXCL1, CXCL2, and CCL20, in a positive feedback way to recruit neutrophils to enhance inflammatory responses [68, 69].

As part of the innate defence against intestinal inflammation, neutrophils are essential for controlling bacterial diseases. Neutrophils engulf and kill bacteria and release an abundant amount of ROS to form a special structure called neutrophil extracellular traps, which are known as critical factors that contribute to resisting bacterial infection [70]. Another aspect of the antibacterial strategy in neutrophils is termed nutritional immunity. The mechanism inhibits the growth of pathogens and reduces the activity of bacterial virulence factors by limiting nutritional metal availability to bacteria, including that of zinc, manganese, iron, and copper [6, 71]. These metals are necessary in enzymes and structural cofactors for the survival of all organisms. Among them, the difficult competition between bacteria and the host over zinc has recently been considered a possible target for new antimicrobial therapies [18, 72, 73].

Upon pathogen attack, the S100A8/S100A9 heterodimer, also known as calprotectin, as one of the most abundant antibacterial proteins in neutrophils, is recruited to the infected site to chelate zinc ions and limit their access to invasive pathogens, further inactivating the superoxide defence of bacteria and inhibiting the growth of *E. coli*, *S. aureus*, *Salmonella* and other microbes [6, 71, 74, 75]. S100A7 (psoriasin) is expressed and acts as an effective chemical barrier in epithelial cells by zinc chelation to inhibit microbial growth [76, 77]. The zinc-binding protein S100A12 (calgranulin C) mostly participates in superoxide formation to exert its antibacterial activity. It also affects ubiquitin and beta-catenin degradation in host cells by interacting with S100A9 (also known as calgranulin B or MRP14) or directly with calcyclin-binding protein and Siah-1-interacting protein [71, 78]. During inflammation, both IL-17 and IL-22 can upregulate the gene expression of S100A8 (calgranulin A, MRP8), S100A9 and S100A12 [79]. When these proteins are released from the cell, they can bind and activate cell surface receptors (Figure 2), such as Toll-like receptor 4 (TLR4), receptor for advanced glycation end-products (RAGE), GPCRs, etc.; initiate intracellular inflammatory signal transduction; and play an important role in the regulation of immune and inflammatory responses [7, 78]. Although S100A9 activates TLR4 to induce inflammation that does not depend on zinc ions, it still acts as a zinc chelator and results in decreased intracellular free zinc levels in the cell [80]. The apoptosis-inducing activity of S100A8/S100A9 is also dependent on both receptor-mediated and zinc exclusion-modulated pathways [81, 82]. Therefore, zinc nutritional immunity is an excellent strategy to fight against bacterial infection in the host.

Recent studies have shown that nociceptors play a role in host defence against STm infection, especially those nociceptors with transient receptor potential cation channel V1 (TRPV1) and tetrodotoxin-resistant voltage-gated sodium channel NaV1.8 expression, decreasing the number of M cells in ileal Peyer’s patches and increasing the colonization of segmental filamentous bacteria, resulting in a reduction in *Salmonella* invasion [11]. This is not a commonly known antimicrobial defence mechanism. As reported, TRPV1-expressing nociceptors can sense bacterial infections, including STm, *C. rodentium* and ETEC, and release calcitonin gene-related peptide (CGRP) or substance P (SP) within the ileum to promote host defence [11, 12, 83, 84]. Elucidation of the complex interactions between intestinal immune cells, neurons, and microorganisms (the “microbe-gut-brain axis”) in mammals will further enhance our understanding of intestinal immunity and host defence [12, 85].

In fact, there are abundant neurons in the gastrointestinal tract. They can detect specific metabolites, cell wall components and toxins of pathogens and play an important role in regulating inflammation, bowel movements and intestinal secretion [12, 85]. Simultaneously, the innate and acquired immune responses of the gastrointestinal tract can be activated or inhibited via the varied stimulation of SP, CGRP, vasoactive intestinal peptide (VIP) and pituitary adenylate cyclase-activating polypeptide released from the enteric nervous system [12]. Nociceptors are the main sensory receptors that respond to gastrointestinal disorders and potentially harmful stimuli, including pathogens and their components, extreme temperature, toxic chemicals, inflammatory mediators, and mechanical and tissue damage, evoking the sensation of pain and improving the body’s resistance during periods of infection [85–87]. The endogenous transient receptor potential (TRP) superfamily of ion channels responds to agonists, stimulates the activity of nociceptive sensory neurons and results in neurogenic inflammation and pain [10]. Intracellular zinc (10 nM) is available for TRP ankyrin subtype 1 protein activation and the subsequent release of inflammatory neuropeptides [10, 88]. Recently, neuropeptides were also reported to be used as a substitute for antibiotics in diarrhoea treatment. For example, VIP treatment can reduce the incidence of diarrhoea and increase the growth rate in newborn weaned piglets. In addition, the increase in inflammatory mediators induced by ETEC infection, including IL-2, IL-12, interferon (IFN)-γ, and TNF-α, and enterotoxin-induced jejunal fluid secretion can be reversed in a dose-dependent manner by VIP treatment [89].

## Zinc affects the activity of host receptors and immune responses

In a recent study, zinc was found to affect the biological activity of host receptors and influence the interaction between pathogenic microbes and the host. For example, porcine aminopeptidase N (APN) is a Zn^2+^-dependent membrane-bound exopeptidase that is widely expressed in various tissues and cells, especially in the small intestine mucosa [90]. It has been proven to be a fimbrial receptor protein for ETEC F4 and mediates the endocytosis of F4 bacteria in the host [91]. In our previous study, we observed that APN directly interacted with the major FaeG subunit of F4 fimbriae and affected F4 bacterial adherence to host cells [92]. The binding determinants of the APN-FaeG interaction contain residues important for zinc binding, and in a certain concentration range, the change in Zn^2+^ concentrations affects the biological activity of the APN protein as well as the adherence between *E. coli* F4 and host intestinal epithelial cells [90, 93]. In addition to cleaving N-terminal amino acids from small oligopeptides on the apical surface of intestinal epithelial cells, APN participates in regulating the MAPK/ERK1/2 signalling pathway in monocytes, while its zinc-binding site is blocked by inhibitory antibodies [91, 94]. APN has also been described as a cancer-specific biomarker and reported to be involved in the progression of tumour metastasis, angiogenesis and prognosis, as well as protein overexpression in cancer cells, while tissue invasion and metastasis in human prostate cancer could be attenuated by using Zn^2+^ to inhibit APN biological activity in a dose-dependent manner [95–97].

Zinc is also closely related to some transmembrane receptor proteins, including B cell receptors (BCRs), TLRs and nucleotide-binding oligomerization domain (NOD)-like receptors, which are responsible for the recognition of microbes or antigen molecules and involved in the development and modulation of intestinal homeostasis [72, 98, 99]. ZIP9 increased the intracellular zinc level and enhanced Akt and Erk phosphorylation in response to BCR activation [100]. ZIP7 plays an important role in B cell development and positively regulates pre-BCR and/or BCR signalling, while ZIP10 deficiency leads to impairment of BCR signalling in immune responses [99, 101, 102]. In addition, zinc induces inflammatory responses via the TLR/nuclear factor kappa-B (NF-κB) pathway and indirectly regulates TLR signalling via zinc transporters [103, 104].

Interestingly, the antimicrobial roles of macrophages vary in defending against different pathogens. Macrophages deploy both zinc starvation and/or zinc toxicity as an antimicrobial strategy [72, 98, 105]. The clearance of *Streptococcus* in macrophages is promoted by both calprotectin-mediated zinc starvation and intracellular zinc toxicity [106], whereas zinc starvation is utilized as a strategy against *Histoplasma capsulatum* infection, and excessive zinc poisoning within the macrophage phagolysosome exerts direct bactericidal effects on *M. tuberculosis* and UPEC [98, 107, 108]. In addition, continuous stimulation of TLRs and NOD2 alters the expression of MT and increases the level of intracellular zinc, leading to increased autophagy and enhanced bacterial clearance of *S.* typhimurium, *S. aureus*, and adherent invasive *E. coli* in intestinal macrophages [98, 109–111].

Zinc also causes macrophage malfunction, further triggering an abnormal inflammatory response, and impairs the innate immune system [111, 112]. For example, IRF/IL-23-mediated M1 macrophage activation and GATA3/IL-4-mediated M2 macrophage inhibition are both aggravated by zinc deficiency, resulting in severe intestinal inflammation and nitrogen metabolism disorder in patients with liver cirrhosis, whereas zinc supplementation can improve ammonia and protein metabolism and regulate the activation of M1/M2 macrophages [113]. Alternative activation of macrophages does not contribute to defending against intracellular pathogens in some cases. IL-4 induces macrophage polarization to the M2 phenotype and increases the expression of MT3 and ZnT4 but not MT1 and MT2. In addition to promoting zinc uptake by intracellular pathogens, MT3 and ZnT4 augments intracellular labile Zn^2+^ and contributes to pathogen persistence in M2 macrophages [114].

Moreover, zinc itself has a regulatory role in intestinal immune responses and the healthy balance of the intestinal flora [3, 108]. After antigen stimulation, zinc attenuates the intestinal stress response and promotes the secretion of proteins and pro-inflammatory cytokines, such as sIgA, antimicrobial peptides, IL-1β, and IL-6 [54]. By regulating the MAPK and Akt signalling pathways, zinc can enhance intestinal mucosal barrier integrity and reduce inflammation and diarrhoea caused by bacterial infections [115]. Zinc also increases the expression of zinc finger protein A20 (also known as tumour necrosis factor alpha-induced protein 3) in intestinal epithelial cells to inhibit the inflammatory response and apoptosis mediated by some signal transduction pathways, such as NF-κB, and subsequent TNF-α production [116, 117]. However, A20 is a potent inhibitor of NF-κB-, TLR3-, and retinoic acid-inducible gene I-mediated IFN induction; thus, along with increased activation of NF-κB, interferon regulatory factor (IRF)-3, and IRF7, viral clearance was improved in A20-deficient cells, and this deficiency may protect the host against viral infection [118, 119].

As reported, zinc could optimize anti-tumour effects by inhibiting LPS-, ROS- or other immune factor-induced oncogenic signalling pathways, such as NF-κB, activator protein-1, Janus tyrosine kinase/signal transducer and activator of transcription (JAK/STAT), and PI3K/Akt; decrease oxidative stress and inflammatory responses induced by chemo- and radiotherapy; promote the development and differentiation of T and B lymphocytes; and improve the tumour microenvironment to reduce the risk of prostate, oesophageal, lung, and oral cancers [120–122]. High dietary zinc treatment has a beneficial effect on the modulation of mucosal structure and immune responses in the gut. It blocked TNF-α-mediated degradation of IκBα, decreased the mRNA expression of IFN-γ and IL-8, and upregulated the mRNA expression of ZO-1, occludin and transforming growth factor-β, resulting in an improvement in the antioxidant capacity and prevention of postweaning diarrhoea in piglets [63, 123].

Additionally, zinc is also reported to influence the modulation of biological rhythm and activity. By effectively stabilizing the binding of period proteins and cryptochrome proteins, zinc ions help to sustain the rhythmicity of the mammalian circadian clock in resisting the interference of negative factors, such as improper diet and drug abuse [124]. Previous studies have shown that zinc can regulate the absorption of water and electrolytes in the intestine and has a general inhibitory effect on voltage-gated ion channels, i.e., those for K^+^, Na^+^, Ca^2+^, and Cl^−^ [125]. A new type of selective chloride channel regulated by Zn^2+^ in enteroendocrine cells of Drosophila larvae’s digestive tract, called Hodor, was found to act as a zinc ion sensor in the intestine and regulate individual feeding behaviour [126, 127]. Zn^2+^ is also used to promote the efficiency of antibiotics, such as vancomycin, quinolones, aminoglycosides, tetracycline, and macrolides [128]. For example, in gram-negative bacteria, the presence of zinc ions leads to an increase in the absorption of quinolones by changing the permeability of the bacterial outer membrane porin OmpF protein, while Zn^2+^ can effectively inhibit the acetylation of aminoglycosides catalysed by aminoglycoside 6′-N-acetyltransferase type Ib [AAC(6′)-Ib] and enhance the sensitivity of bacteria, especially for some aminoglycoside-resistant bacteria [128, 129].

When zinc deficiency occurs, the cytotoxicity of natural killer cells, the phagocytic activity of neutrophils and the chemotaxis of monocytes are significantly decreased, but the phagocytosis and oxidative burst of monocytes are not affected. Zinc shortage has an influence on only IL-6 and TNF-α production in monocytes [54, 112]. In addition, the loss of copper-zinc superoxide dismutase activities results in damage to the stability of the cell membrane structure and dysfunction in host defensive systems, such as the regulation of neutrophil apoptosis and neutrophil-mediated tissue injury [117]. Because of this dysfunction, zinc deficiency enhances the severity of infectious diseases and illness in animals, whereas probiotics were reported to overcome the host defence defect in zinc-deficient cells by abolishing the activation of pro-inflammatory signalling via the ERK and p38 pathways [130].

## Zinc is vital for viral infections

It is known that the host is more vulnerable to enterovirus or toxigenic bacterial infections in zinc-limited conditions, while those pathogens both stimulate chloride secretion by activating intracellular guanosine monophosphate and adenylate cyclase [131]. During this period, poor nutritional absorption and diarrhoea further aggravate the compromised mineral state, and zinc supplementation can attenuate this damage [132, 133]. Thus, zinc plays an important role in both bacterial and viral infections.

Indeed, zinc was previously considered a potential agent for the prevention of H1N1 influenza (“swine flu”) and has direct antiviral properties [134]. Studies have shown that the replication of severe acute respiratory syndrome coronavirus (SARS-CoV) and hepatitis C virus (HCV) was inhibited by zinc oxide or zinc salt, while zinc could be deposited onto the surfaces of herpes simplex virus (HSV) virions, and the viral ubiquitin–proteasome pathway could be targeted with the zinc ionophore pyrithione to inhibit HSV-1 and HSV-2 infections [14, 135–137]. Upon exposure to low endosomal pH, zinc binding with a specific histidine residue on the viral E1 protein can effectively inhibit membrane fusion and block particle release of respiratory syncytial virus, HSV, Semliki Forest virus, and sindbis viruses [138, 139]. Moreover, zinc concentration is positively correlated with MT synthesis [54], which is an abundant zinc-binding protein and has a selective antiviral effect against viruses. MT acts as a zinc chaperone, promotes antiviral signal transduction and interferes with a potent antiviral response by modulating zinc influx or redistribution. Influenza, human immunodeficiency virus (HIV), HCV, measles virus, human cytomegalovirus and coxsackievirus infections are associated with upregulated MT expression, leading to a higher NF-κB DNA binding affinity [139, 140]. In the case of HIV, a significant increase in intracellular zinc and MT expression contributed to the inhibition of HIV-infected monocyte apoptosis by suppressing caspase-3 activation, which is conducive to the persistence of HIV infection [141, 142].

Thus, as a recent review summarized, the antiviral effects of zinc can be separated into two major categories. First, virus replication or infection-related symptoms are specifically inhibited or attenuated by zinc treatment. Second, zinc supplementation has protective effects against viruses by boosting the antiviral and systemic immunity of patients with zinc deficiency [108, 139]. In addition, zinc is an integral structural element of many viral proteins, including enzymes, proteases, and polymerase, and is thus crucial for virus transmission, as well as innate and adaptive antiviral responses [139, 143]. The protective role of zinc against coronavirus disease 2019 (COVID-19, caused by SARS-CoV-2) might be a good example to understand this effect. From the outset, zinc directly inhibits RNA-dependent RNA polymerase activity to block viral replication, such as that of SARS-CoV, equine arteritis virus (EAV), hepatitis E virus, etc. Both chloroquine (CQ) and its derivative hydroxychloroquine depend on this strategy to prevent the replication of SARS-CoV-2 in host cells [13, 144, 145]. CQ also induces the uptake of zinc, which results in an increase in cellular zinc accumulation and a lower risk of secondary infection, especially for patients with viral pneumonia coinfected with *Streptococcus pneumoniae* (*S. pneumoniae*) since zinc inhibits the growth of *S. pneumoniae* by regulating bacterial Mn(II) homeostasis [13, 144, 146].

Apart from the direct antiviral and antibacterial effects mentioned above, zinc is essential to ameliorate SARS-CoV-2-induced lung injury and other worse outcomes. For instance, zinc represents a promising agent to preserve the barrier function of the respiratory epithelium; it functions as a potent antioxidant and promotes ciliary beat frequency, mucociliary clearance, and ZO-1 and claudin-1 expression [13, 147, 148]. In a recent study, zinc appeared to affect the entry of SARS-CoV-2 into cells by decreasing the activity of angiotensin-converting enzyme 2, which is a receptor that binds primarily to the spike protein on the surface of coronaviruses [13, 149]. Zinc also has a critical role in resisting COVID-19. It degrades viral RNA and inhibits gene translation by various antiviral mediators, such as ribonuclease L and RNA-dependent protein kinase, which is induced by IFN-α via the JAK/STAT pathway [144, 150]. In view of the fact that severe COVID-19 frequently occurs along with pulmonary fibrosis and septic shock, a deficiency in zinc intake under the condition of sepsis may further increase the incidence and severity of COVID-19, and zinc supplements may stave off LPS-induced neutrophil recruitment into the lung and thus alleviate lung injury in systemic inflammation [13, 147]. In addition, zinc also minimizes the risk of excessive release of inflammatory cytokines (“cytokine storm”) by inhibiting IKK activity and subsequent NF-κB signal transduction, as well as regulatory T cell functions [146, 151].

## Conclusions

In recent decades, host hormones and QS of bacteria were found to be effective regulators of crosstalk between bacteria and their host [16, 17]. The inter-kingdom signalling between them provides a new way to understand microbial infections and host immunity. Likewise, zinc serves as an important agent for the development of cell structure and functions. It may promote the survival and virulence of bacteria, viruses, or other microbes and participate in host defence by modulating various modes of signal transduction and inflammation in a concentration-dependent way [5, 22, 72, 98].

Both zinc deficiency and excess zinc toxicity can be used as a strategy to resist microbial infections. Upon stimulation, due to the variety of zinc sensors and channels distributed in different regions or tissues in the body, zinc may have an adverse effect on host immune regulation in different cells. Thus, zinc homeostasis in cells must be strictly regulated. In this review, we also discussed a recent study on zinc competition between hosts and microbes, inflammation and the immune response modulated by zinc-mediated nociceptive sensory neuron-immune interactions, and the change in host receptors with zinc treatment during infection, which provides a basis to clarify pathogenesis, representing a profound impact on our understanding of host defence and a promising target for new antimicrobial strategies. However, the molecular mechanism of zinc in the interplay between the host and microbes still needs to be elucidated.

## Data Availability

Not applicable.

## Abbreviations

APN: Aminopeptidase N
CDF: Cation diffusion facilitator
CGRP: Calcitonin gene-related peptide
COVID-19: Coronavirus disease 2019
*C. rodentium*: *Citrobacter rodentium*
DMT1: Divalent metal transporter 1
EAV: Equine arteritis virus
ER: Endoplasmic reticulum
ETEC: Enterotoxigenic *E. coli*
UPEC: Uropathogenic *E. coli*
GPCRs: G protein-coupled receptors
GPR39: G protein-coupled receptor 39
HCV: Hepatitis C virus
HIV: Human immunodeficiency virus
HSV: Herpes simplex virus
IL: Interleukin
IFN: Interferon
IRF: Interferon regulatory factor
MAPK: Mitogen-activated protein kinase
MT: Metallothionein
mTOR: Mammalian rapamycin target protein
*M. tuberculosis*: *Mycobacterium tuberculosis*
NHE: Na^+^/H^+^ exchanger
NOD: Nucleotide-binding oligomerization domain
QS: Quorum sensing
RAGE: Receptor for advanced glycation end-products
RND: Resistance nodulation division
Pit: Inorganic phosphate transport
PI3K: Phosphatidylinositol 3-kinase
p70S6K: P70 ribosomal S6 protein kinase
PKCz: Protein kinase C-ζ
RdRp: RNA-dependent RNA polymerase
ROS: Reactive oxygen species
*S*. *aureus*: *Staphylococcus aureus*
SARS-CoV: Severe acute respiratory syndrome coronavirus
sIgA: Secretory immunoglobulin A
SP: Substance P
*S. pneumoniae*: *Streptococcus pneumoniae*
STm: *Salmonella* Typhimurium
TJ: Tight junction
TLR4: Toll-like receptor 4
TNF-β: Tumour necrosis factor β
TRPV1: Transient receptor potential cation channel V1
VIP: Vasoactive intestinal peptide
ZO-1: Zonula occludens-1
